# Protease inhibitor GC376 for COVID-19: Lessons learned from feline infectious peritonitis

**DOI:** 10.1016/j.amsu.2020.12.030

**Published:** 2020-12-28

**Authors:** Khan Sharun, Ruchi Tiwari, Kuldeep Dhama

**Affiliations:** a Division of Surgery, ICAR-Indian Veterinary Research Institute, Izatnagar, Bareilly, 243 122, Uttar Pradesh, India; b Department of Veterinary Microbiology and Immunology, College of Veterinary Sciences, Uttar Pradesh Pandit Deen Dayal Upadhyaya Pashu Chikitsa Vigyan Vishwavidyalaya Evam Go Anusandhan Sansthan (DUVASU), Mathura, 281001, India; c Division of Pathology, ICAR-Indian Veterinary Research Institute, Izatnagar, Bareilly, 243 122, Uttar Pradesh, India

**Keywords:** COVID-19, SARS-CoV-2, Therapeutics, Feline infectious peritonitis, Feline coronavirus, Main protease inhibitor

## Abstract

The main protease (M^pro^) of severe acute respiratory syndrome coronavirus 2 (SARS-CoV-2) is an important therapeutic target as it plays a major role in the processing and maturation of the viral polyprotein. GC376 is a pre-clinical dipeptide-based protease inhibitor that has been previously used for managing feline infectious peritonitis virus (FIPV). Since both GC373 and GC376 have already been successfully used in treating animal coronavirus infection, they can be considered as strong drug candidates for COVID-19 in humans. GC376 is a broad-spectrum antiviral drug that inhibits M^pro^ of several viruses, including the coronaviruses like feline coronavirus, porcine epidemic diarrhoea virus, severe acute respiratory syndrome coronavirus, Middle East respiratory syndrome coronavirus, ferret, and mink coronavirus. However, further studies should be conducted to evaluate the potency, efficacy, and safety of these broad-spectrum M^pro^ inhibitors in patients with COVID-19. The lessons learned from the successful use of drug candidates for treating animal coronavirus infections will help us to develop framework for their use in human trials.

Coronavirus disease 2019 (COVID-19) has affected millions of people around the world and has resulted in more than a million deaths. It is caused by severe acute respiratory syndrome coronavirus 2 (SARS-CoV-2), a novel zoonotic coronavirus that was first reported in Wuhan, Hubei province, China, in December 2019 [1]. The main protease (M^pro^) of SARS-CoV-2, also called as 3CL^pro^, is an important therapeutic target due to its important role in the processing and maturation of the viral polyprotein [2,3]. GC376 is a pre-clinical dipeptide-based protease inhibitor, used against feline infectious peritonitis virus (FIPV), a strain of feline coronavirus (FCoV) [2,4]. Infection with FCoV is associated with only mild symptoms, but can lead to feline infectious peritonitis (FIP) that can be fatal in cats [5]. This provides an important perspective on the pathogenic aspects of COVID-19 that are yet to be understood [6,7]. Although FCoV and SARS-CoV-2 are taxonomically distant and distinct, some of the pathogenic and immunopathogenic characteristics observed in the cats with FIP seem to be present in the patients with COVID-19 [7].

Coronaviral infections in animals have been widely studied for decades especially the unique involvement of central nervous system (CNS) in cats with FIP [8,9]. A similar scenario is uprising in the case of SARS-CoV-2 infection in human beings wherein there is an increase in neurological manifestations that is expected to be the result of direct CNS involvement [8]. The adenosine nucleoside analogue GS-441524 is the main plasma metabolite and the active form of Remdesivir (GS-5734), an antiviral drug developed by Gilead Sciences [6,7]. It is considered to be one of the most promising, direct-acting antiviral drugs against FIP [7]. Therefore, GS-441524 can be another therapeutic option for managing COVID-19 infection [6,7]. GS-441524 has already exhibited promising results for treating non-neurological FIP. However, studies evaluating the efficacy of GS-441524 in managing neurological FIP are limited. Dickinson et al. (2020) reported that all the cats with neurological FIP responded positively when treated with GS-441524 (5–10 mg/kg) for a minimm of 12 weeks [9]. However, the dose of GS-441524 used was higher than that used for non-neurological FIP. One of the characteristic MRI findings observed in neurological FIP is the multifocal leptomeningeal lesions (Fig. 1) [8]. Although the incidence and mechanisms of CoV infection as well as the CNS pathology varies, the lessons learned from treating infection within the CNS will be useful to manage COVID-19.

**Fig. 1:**
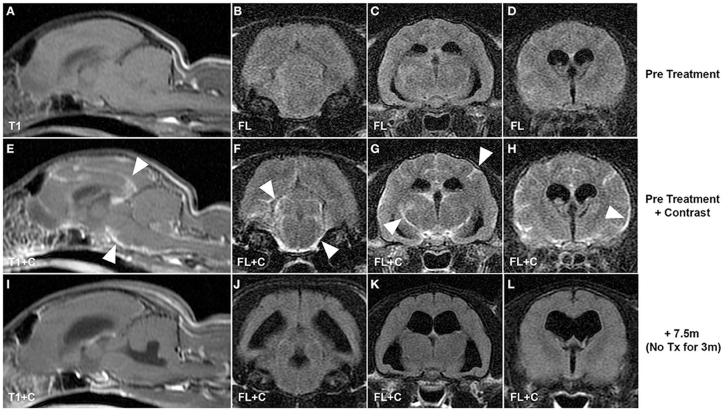
Neurological FIP in a cat with CNS involvement presented with neurological deficits that was treated with GS-441524. Reproduced from Dickinson (2020) Creative Commons Attribution License (CC BY). A–D: pre-contrast, pre-treatment MRI sequences. E–H: post-contrast T1-weighted and fluid-attenuated inversion recovery MRI sequences showing multifocal leptomeningeal lesions (arrowheads). I–L: treatment with GS-441524 (10 mg/kg) resulted in resolution of clinical signs and MR lesions on images acquired 7.5 months after initiation of treatment. (T1 - T1-weighted, FL - fluid-attenuated inversion recovery, +C - using contrast: gadopentetate dimeglumine).

The safety and efficacy of GC376, a dipeptide-based protease inhibitor was previously evaluated on client-owned cats with FIP, where it has showed promising therapeutic efficacy, particularly in the cats with certain presentations of FIP [4]. It also inhibits SARS-CoV-2 in Vero cells by targeting the catalytically active sites of M^pro^ [2], and has antiviral activity against SARS-CoV-2 at an EC_50_ value of 3.37 μM [10]. In addition, it acts against SARS-CoV and MERS-CoV, the other two zoonotic coronaviruses infecting human beings [3,11]. Studies have also shown that it can inhibit the main protease of ferret and mink coronavirus [12]. Therefore, GC376 can be considered as a broad-spectrum antiviral drug that inhibits M^pro^ of several viruses, including the coronaviruses like FCoV, porcine epidemic diarrhoea virus (PEDV), SARS-CoV, MERS-CoV, SARS-CoV-2, ferret, and mink coronavirus [3,5,11,12]. This may be because of the highly conserved structure of M^pro^ among these viruses [5,12].

GC376 is the prodrug of GC373, another dipeptide-based protease inhibitor. In addition to being the prodrug, GC373 was also reported to effectively inhibit the M^pro^ of SARS-CoV-2 with an IC_50_ value in the nanomolar range [5]. The ability of GC373 and GC376 to inhibit SARS-CoV-2 was evaluated with plaque reduction assays using infected Vero E6 cells. The findings indicate that both the drugs are efficient inhibitors of SARS-CoV-2 with high therapeutic index (>200) [5]. Based on the available data, both GC373 and GC376 can be advanced quickly into the next stage of evaluation that includes human trials.

Although the RNA-dependent RNA polymerase (RdRp) of SARS-CoV-2 has proof-reading function, the virus mutates, leading to drug resistance [13]. SARS-CoV-2 M^pro^ inhibitors can be used either alone or in combination with viral RdRp-inhibitors, to achieve synergistic antiviral activity and to suppress the emergence of drug resistance [10]. The combined use of GC376 with Remdesivir, a nucleotide analogue that inhibits RdRp of SARS-CoV-2, produces additive effect, thereby enhancing the overall antiviral activity [2]. Remdesivir monotherapy has raised concerns regarding the possible development of drug resistance [13]. Therefore, the addition of SARS-CoV-2 M^pro^ inhibitors, such as GC376, into the treatment regimen will ensure that SARS-CoV-2 infection is controlled at multiple levels (Fig. 2).

**Fig. 2:**
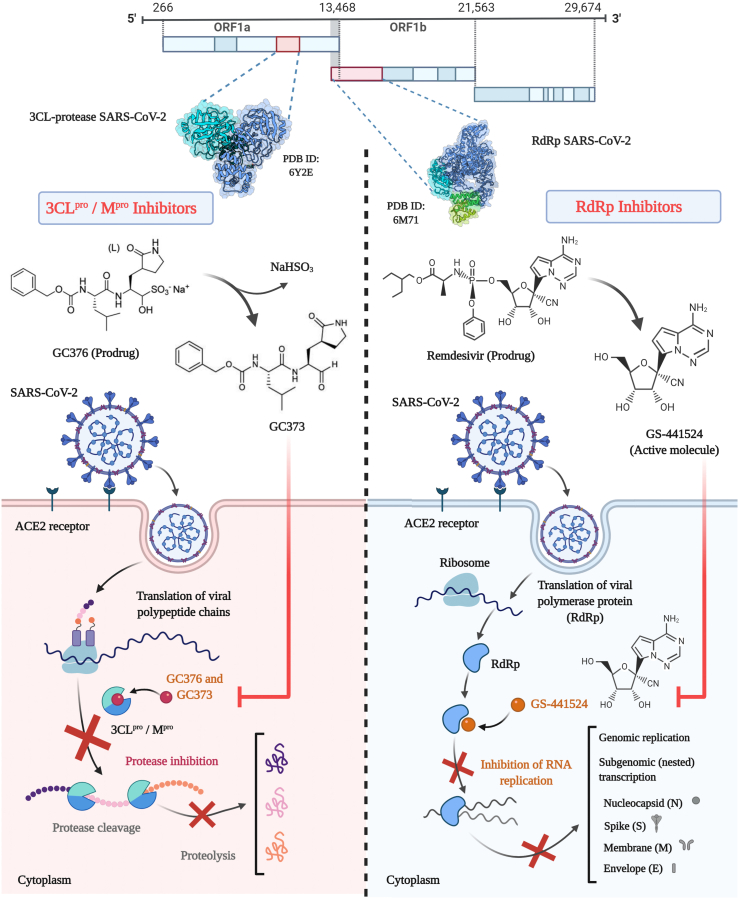
Therapeutic candidates that can inhibit the replication of SARS-CoV-2 by inhibiting the main protease (M^pro^) (also called as 3CL^pro^) and RdRp.

Treatment of FIP in cats with GC376 was associated with side effects such as transient stinging at the injection sites, subcutaneous fibrosis, hair loss, and abnormal eruption of permanent teeth in juvenile cats [4]. Therefore, further studies are required to evaluate the possible side effects associated with the use of GC376 in animal models, before its use in clinical trials. Considering its potentials for side effects, GC376 should only be used for a short-period (1–2 weeks) to treat COVID-19 [2].

## Conclusion and future perspectives

SARS-CoV-2 M^pro^ is an important drug target as it plays an essential role in the cleavage of viral polypeptides. Repurposed antiviral drugs, especially the protease inhibitors can be considered as an important therapeutic strategy for managing COVID-19. Since both GC373 and GC376 have already been successfully used in treating animal coronavirus infection, they can be considered as strong drug candidates for COVID-19 in humans. Both the drugs inhibit the replication of SARS-CoV-2 in cell cultures by inhibiting M^pro^. Therefore, further studies can be conducted to evaluate the potency, efficacy, and safety of these broad-spectrum SARS-CoV-2 M^pro^ inhibitors in patients with COVID-19. Over the years, researchers have tried different strategies to develop or identify suitable therapeutic candidates against FIPV. The experience they have gained through these studies is now becoming fruitful in identifying therapeutic drugs for COVID-19. The lessons learned from the successful use of drug candidates for treating animal coronavirus infections will help us to develop framework for their use in human trials. Furthermore, understanding the mutation that give rise to virulent and lethal FIPV will provide an insight into the relationship between different strains of SARS-CoV-2 and their virulence.

## Funding

No substantial funding to be stated.

## Provenance and peer review

Not Commissioned, internally reviewed.

## Declaration of competing interest

All authors declare that there exist no commercial or financial relationships that could, in any way, lead to a potential conflict of interest.
